# Contribution of the basal forebrain to corticocortical network interactions

**DOI:** 10.1007/s00429-021-02290-z

**Published:** 2021-05-22

**Authors:** Peter Gombkoto, Matthew Gielow, Peter Varsanyi, Candice Chavez, Laszlo Zaborszky

**Affiliations:** 1 Center for Molecular and Behavioral Neuroscience, Rutgers University, 197 University Avenue, Newark, NJ 07102 USA; 2 ETH Zurich Institute of Neuroinformatics, 8057 Zurich, Switzerland

**Keywords:** Basal forebrain, Orbitofrontal cortex, Visual association cortex, Gamma coherence, Optogenetics, High-density recordings

## Abstract

Basal forebrain (BF) cholinergic neurons provide the cerebral cortex with acetylcholine. Despite the long-established involvement of these cells in sensory processing, attention, and memory, the mechanisms by which cholinergic signaling regulates cognitive processes remain elusive. In this study, we recorded spiking and local field potential data simultaneously from several locations in the BF, and sites in the orbitofrontal and visual cortex in transgenic ChAT-Cre rats performing a visual discrimination task. We observed distinct differences in the fine spatial distributions of gamma coherence values between specific basalo-cortical and cortico-cortical sites that shifted across task phases. Additionally, cholinergic firing induced spatial changes in cortical gamma power, and optogenetic activation of BF increased coherence between specific cortico-cortical sites, suggesting that the cholinergic system contributes to selective modulation of cortico-cortical circuits. Furthermore, the results suggest that cells in specific BF locations are dynamically recruited across behavioral epochs to coordinate interregional cortical processes underlying cognition.

**Supplementary Information:**

The online version contains supplementary material available at 10.1007/s00429-021-02290-z.

## Introduction

The basal forebrain (BF) corticopetal projection system is the main source of acetylcholine (ACh) for all neocortical areas. BF areas rich in cholinergic neurons also contain GABAergic and glutamatergic corticopetal projection neurons, as well as various interneuron cell types (Gritti et al. 2003; Zaborszky and Gombkoto 2018). This anatomically complex system has been implicated in cortical activation, affect, attention, sensory coding, motivation, and memory. Lesions or blockade of ACh in the cortex results in impairments in perception (Minces et al. 2013), cognitive flexibility (Prado et al. 2017), executive function, and cortical plasticity (Conner et al. 2003, 2010; Ballinger et al. 2016). Evidence from tracing studies and lesions has suggested that the cholinergic projection system is organized topographically: for example, posterior lesions of BF produce more damage to the cholinergic innervation of the auditory cortex compared to anterior regions (Chavez and Zaborszky 2017). Early functional data, however, have contributed to the view that the cholinergic signaling in the cortex is a slow, non-specific one, most likely acting through volume transmission (Hasselmo 2006; Sarter 2007; Parikh et al. 2007), and that the BF cholinergic projections are part of the “diffuse cortical projection systems” (Saper 1987). Recent anatomical studies paint a more complex picture, wherein the cholinergic projection to the neocortex is not diffuse, but instead is organized into cortical target-specific groups of cholinergic neurons that receive specific combinations of inputs (Zaborszky et al. 2015; Gielow and Zaborszky 2017; Chavez and Zaborszky 2017). Moreover, cholinergic cells that project to the superficial or deep layers of the medial prefrontal cortex (mPFC) in mice are largely separated in the BF (Bloem et al. 2014). Also, new evidence from real-time amperometric recording indicates cholinergic signaling in attentional contexts that is rapid, phasic, transient, and probably synaptic (Sarter and Lustig, 2020). Based on the suggestion that the organization of projections from the BF may enable parallel modulation of multiple groupings of interconnected yet nonadjacent cortical areas (Zaborszky et al., 2015), the aim of the current study is to elucidate the mechanism by which cortico-cortical interactions may be modulated by the BF.

The introduction of multi-electrode arrays (silicon probes) in awake, behaving rodents have begun to unravel the behavioural correlates of BF neurons, although the transmitter character of the recorded neurons was initially elusive (Zaborszky and Gombkoto 2018). For example, a tonic-firing subset of BF neurons burst transiently as an ensemble (Lin et al. 2006), and their synchronization phase was tightly associated with prefrontal (PFC) oscillation power in both the low (< 10 Hz) and gamma range (30–100 Hz). The firing of these putative non-cholinergic neurons was correlated with the salience of reward- or punishment-predicting stimuli, irrespective of their sensory properties. Another recent study recorded spontaneous population activity in the BF and auditory cortex (Yague et al. 2017); however, their BF probe was not implanted in the region containing most of the auditory-projecting cholinergic neurons (Chavez and Zaborszky 2017). Based on the rhythmic firing of a large percentage (45.5%) of neurons mainly at slow frequencies (< 6 Hz), Yague et al. (2017) suggested that BF neurons slowly modulate cortical neurons. In contrast to these studies, the group led by Chiba and Nitz (Tingley et al. 2014, 2015, 2018), using a large dataset of BF neuronal activity (*n* = 1428), suggested that the firing rate dynamics of populations of BF neurons oscillate in a nested fashion (“multiplexing”) at different frequencies, including theta, beta, low and high gamma. These latter authors further suggest that this multiplexing maximizes information transfer from the BF to those cortical regions relating to working memory, reward encoding, motor states, and decision making.

**Fig. 1:**
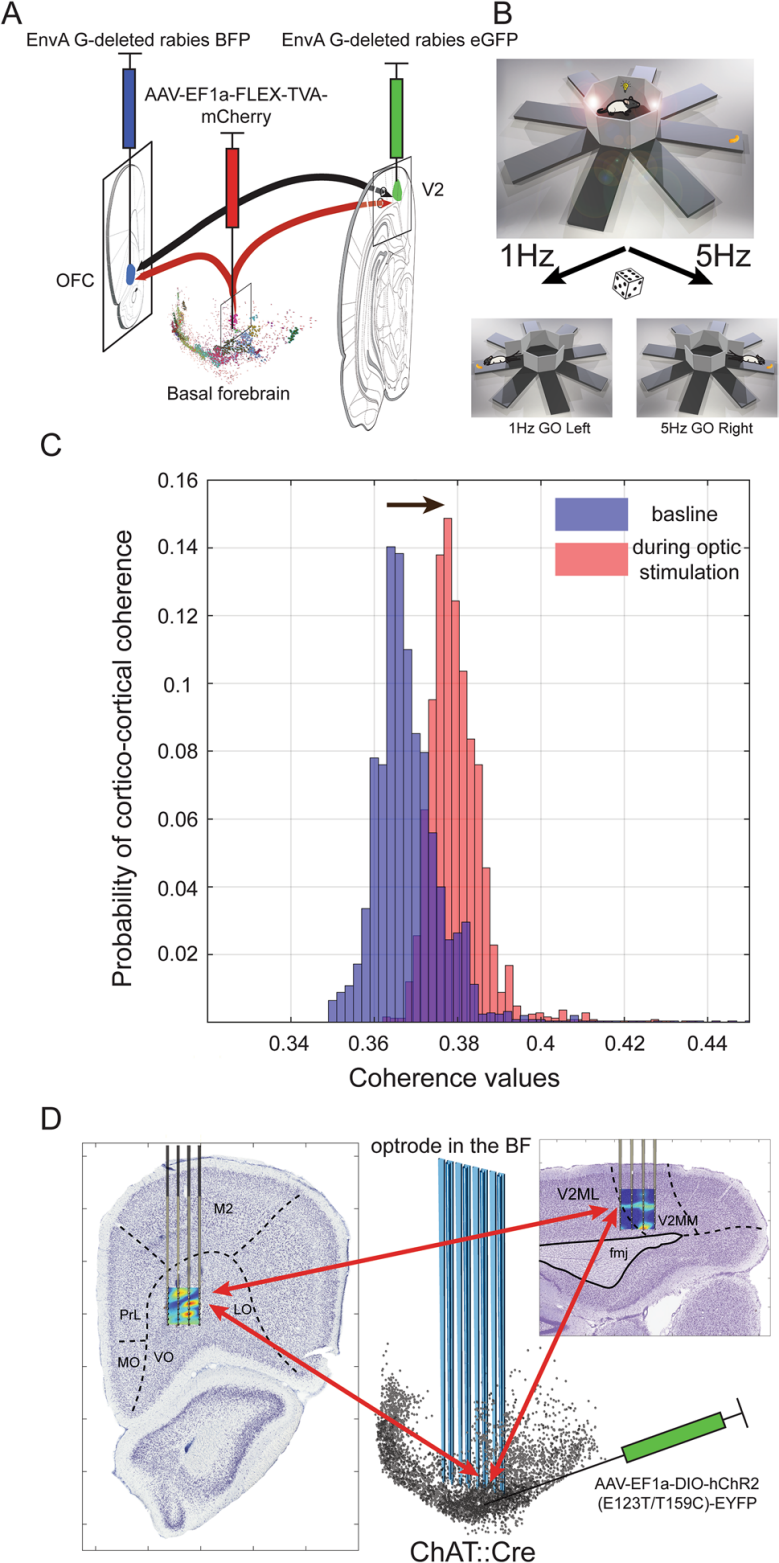
Experimental paradigm. **a** To study cholinergic projections to V2 and OFC, one animal received a helper virus in the ipsilateral BF as described (Gielow and Zaborszky 2017), followed by EnvA G-deleted rabies BFP injected in the OFC and EnvA G-deleted rabies eGFP into the V2 cortex. **b** Visual discrimination task. For the discrimination task, radial arm maze was used utilizing only 2 arms. The doors were closed until a flashing light cue occurs on both doors at two different frequencies (1 Hz left, 5 Hz right) and then both doors opened. **c** Demonstration of the probability distribution of cortico-cortical coherence between OFC (Channel#2) and V2 (Channel#124). The blue histogram represents the coherence probability of the baseline. Red histogram represents the cortico-cortical coherence values during optic stimulation of cholinergic cells. Black arrow shows the shift of the cortico-cortical coherence by cholinergic activation. **d** Schematic position of silicon electrode array (blue lines with optic fibers attached at alternate shanks) within the cholinergic system (black dots in middle panel). Left: coronal section showing the position of silicon probes in ventral (VO) and lateral orbital (LO) cortex superimposed with local heat map of gamma coherence during decision making; Right: probes in V2 on a sagittal section superimposed with a heat map of local maxima of gamma coherence values during cue presentation. The red arrows represent the conceptualized functional loop between the structures

Given the anatomical inhomogeneity of the BF, studies using single-unit recordings that do not systematically sample throughout the BF (Lin and Nicolelis 2008; Tingley et al. 2015) do not directly address the question of whether the BF modulates disparate cortical regions globally or in a selective fashion. Here, we use silicon probes in awake behaving rats (Fig. 1) to examine the coherence of the basalo-cortical and cortico-cortical networks (Fig. 1c–d), and the role of optogenetically stimulated cholinergic neurons in this process. With this, we are able to record and analyze electrophysiological datasets of meso- to large-scale networks that were previously described only in anatomical studies. Extracellular spikes and LFPs (local field potentials) were recorded simultaneously using 64-contact multi-shank probes chronically implanted in the BF; in addition, two 32-contact multi-shank neuronal probes were implanted in the orbitofrontal (OFC) and visual association cortex (V2) (Fig. 1d). Based on anatomical (Zaborszky 2002) and recent functional studies showing coordinated ACh release (Teles-Grilo Ruivo et al. 2017), we also hypothesized that V2 and OFC areas, which are reciprocally interconnected (Reep et al. 1996), may be jointly modulated by the same BF neurons, including cholinergic cells.

## Materials and methods

Viral tracing was used to reveal cholinergic projections from BF to their cortical (OFC, V2) targets. To compare neuronal activity between multiple cortical and BF subregions, we performed in vivo freely moving electrophysiological experiments in rats. Multi-electrode single-unit spike activity and LFPs were recorded simultaneously from different cortical layers of OFC, V2, and different compartments of the BF in three rats during successfully-learned visual cue discrimination. Supplementary Figure S1 shows an example recording during a single behavioral trial, displaying continuous wavelet transformation (CWT) of LFPs and population firing across all three brain regions, including acceleration of the animal in different behavioral epochs (Fig. S1g). The electrode array sampled across the entire BF, including the ventral pallidum (VP), substantia innominata (SI), horizontal limb of the diagonal band (HDB), and globus pallidus (GP), but excluding medial septum (MS) regions and the most posterior part of the BF.

### Animals

Animals were treated in accordance with the National Research Council’s “Guide for the Care and Use of Laboratory Animals” and with the approval of the Rutgers University Institutional Animal Care and Use Committee. ChAT::Cre rats expressing Cre under the ChAT promoter (Witten et al. 2011), a gift from Dr. Karl Deisseroth at Stanford University, were backcrossed with wild-type Long-Evans rats (Harlan). Three naive ChAT::Cre adults (5+ months of age) were used for the in vivo extracellular electrophysiology experiment; a fourth rat was used only to test the recording and optical stimulation system. Additionally, one animal was used for tracing cholinergic projections to V2 and OFC in ChAT::Cre rat. All rats were housed in cages of one to three animals on a 12-h-light/12-h-dark cycle.

### Viruses and surgeries

**Fig. 2:**
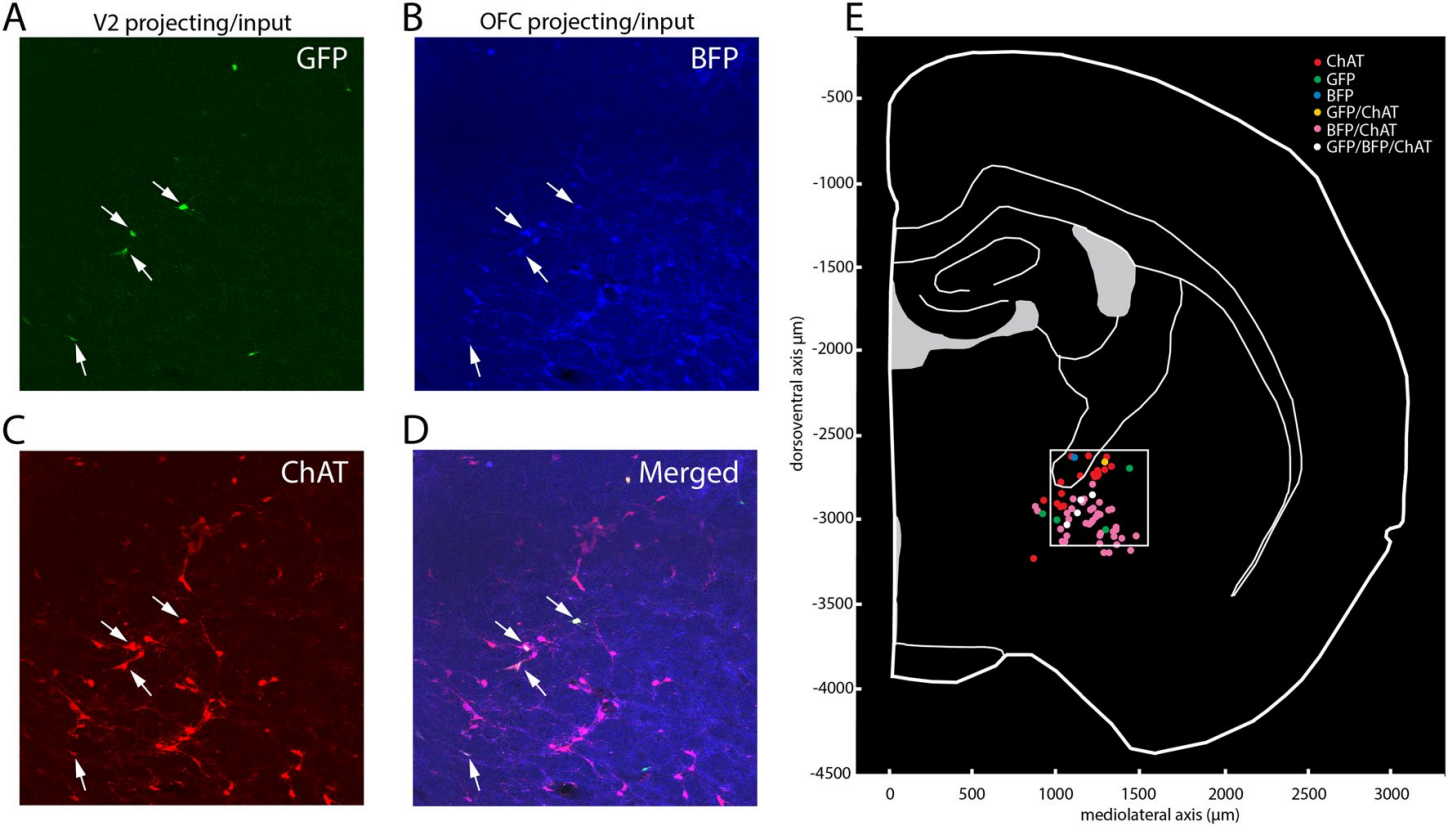
Cholinergic (ChAT) projection neurons to V2 and OFC. **a** V2 projecting GFP labeled neurons, **b** BFP labeled neurons projecting to OFC, **c** mCherry-labeled cholinergic neurons, **d** merged images from (**a**–**c**). Arrows, point in each panel to triple-labeled neurons that is cholinergic and collateralized to V2 and OFC, **e** schematic figure to show the distribution of differently labeled cells in this field of view. Explanation of various color-tagged cells is indicated in upper right. Please note that single green, or single blue cells represent input cells to cholinergic neurons projecting to V2 or OFC, respectively

Subjects were anesthetized with 1–4% isoflurane inhalation in O2. Rats received an intracranial injection of 2.64 µL virus (University of Pennsylvania: AAV-EF1a-DIO-hChR2(E123T/T159C)-EYFP Serotype 5, titer ≥ 1 × 1013 vg/mL)  using a Nanoject-II (Drummond Scientific) via micropipette across five locations covering the right BF (coordinates in mm relative to bregma, from pia, + 0.5 AP, 1.05 ML, 6 DV; 0.0 AP, 1.65 ML, 6.5DV; 0.87 AP, 2.3 ML, 5.2 and 6.5 DV; 1.7 AP, 3.4 ML, 5.6 DV). In order to study cholinergic projections to V2 and OFC, one animal received helper virus (AAV-EF1a-FLEX-TVA-mCherry and AAV-CA-FLEX-RG) in the ipsilateral BF as described (Gielow and Zaborszky 2017), followed by EnvA G-deleted rabies BFP injected in the OFC and EnvA G-deleted rabies eGFP into the V2 cortex; The coordinate for OFC was 4.2 AP; 2 and 1.25 ML, DV 4.2, and for V2 -7.08 AP, 2 ML, 1 DV (Fig. 1a). Blue and red signals were enhanced (Fig. 2) with BFP (N0502-At647N-S FluoTag-X2, ATTO 647N-labeled), and RFP (N0401-SC3-S RFP FluoTag-Q, Sulfo-Cyanine 3-labeled) antibodies. Antibodies were diluted 1:500 in 0.1 M PB (pH 7.4), 3% normal goat serum and 0.1% triton. Sections were incubated for 3 h at room temperature, mounted on slides and cover slipped with DPX (VWR product code: 360294H). Sections were incubated for 3 h at room temperature, mounted on slides and cover slipped with DPX (VWR product code: 360294H). Forebrain sections (*n* = 23, 200 µm series) were viewed by an upright confocal microscope (BX61WI, Olympus). The three channels used: GFP—excitation 488, emission filter 500–545; mCherry—excitation 559, emission filter 575–675; BFP—excitation 405; emission filter 425–475. Panel (d) in Fig. 2 is an image merged by Adobe Photoshop^R^ with no changes in the contrast or brightness.

### Behavioral training

Behavioral training was conducted in a radial arm maze utilizing only 2 arms 180 degrees apart (Fig. 1). The central doors were closed until a flashing light cue (4 s duration) occurred on one of the doors at one of two different frequencies: 1 Hz indicating reward on the left arm, 5 Hz indicating reward on the right arm (Fig. 1b). Next, the two doors were opened simultaneously, and the reward (cheesy poof by Cheetos^®^) was presented at the end of the correct corresponding arm. The reward was not visible to the rat from the gate because the gate was closed when the reward was delivered, in a 1 cm high and 2 cm diameter container at the end of the arm. After consumption of the reward, the rats freely returned to the start position at the center of the maze, and the gates were closed. The rats then waited for the next light cue for the location of the next reward. The cue light/reward location alternated sequentially until the rats got 90% correct, after which the trials were randomly intermixed. Each trial duration was verified manually based on accelerometer data and head position. The behavioral epochs were determined based on the location and acceleration of the rat and divided into 6 different time periods: center/resting, cue presentation (4 s duration), decision making (between door opening and initiation of arm approach), reward approach (during running until the animal stopped at the end of the arm to collect reward; acceleration signals flattened), reward consumption, and return period (running from the end of the arm to return to the center).

### Chronic implantation of probes

Four to twelve weeks after virus injection, the rats were prepared for chronic recording as described in (Royer et al. 2010). Three animals were implanted with a high density silicon probe in the BF with 64 recording sites on eight shanks (8 recording sites on each shank) with the “Buzsaki” V-shape site separation; every second shank had an optic fiber. For cortical regions, we used 32 recording sites on four shanks (8 recording sites on each shank) with 100 µm vertical site separation (Fig. 1d). After recording, histological reconstruction was applied to determine the putative location of the shanks in 3D space (Fig. 1d). Two small skull screws implanted above the cerebellum served as reference and ground electrodes. After enlarging the hole used for the virus injection, dura mater was removed. An opto-silicon probe (Buzsaki64L-H64LP_30mm by Neuronexus) for BF and a linear probe (A4x8-5mm-100-200-177-H32_21) assembly for cortex were attached to micromanipulators and positioned so that the shanks avoided puncturing large vasculature and were inserted into the brain. The visual cortex silicon electrode was positioned parallel to the median sagittal axis {Bregma: from − 6.12 to − 5.32 ML: 2 mm, maximum DV 1.4 mm}. The OFC electrode was positioned coronally {Bregma: 4.2 mm ML: 1.2–2 mm, DV: 3.5–4.3 mm}. In total, 45 recording sessions were performed in three animals (11, 18, and 16 sessions individually). A silicone gel (Dow Corning 3-4680 Krayden -DC4027868) was applied in the hole around the shanks to protect the cortical surface and prevent leakage of CSF. Prior to waking, subjects were given Buprenorphine (0.04 mg/kg, subcutaneous) and Meloxicam (1 mg/kg subcutaneous) to prevent pain and inflammation. The micromanipulator (custom 3D-printed electrode micro-drivers) and a 3D printed cap with copper mesh shield were cemented to the skull around the entire assembly to protect and electrically shield the electrodes while tightly holding the connectors.

### Recording procedures

One week following implantation, rats were food-deprived and trained to run on a radial arm maze that they were previously acclimated to. Extracellular broadband (0.33–5000 Hz) signals were recorded simultaneously from all implanted probes. Recordings occurred daily for approximately one hour until recordings were no longer viable, indicating the endpoint. For the duration of the recording session, a digital head stage was connected to the ‘probes’ output connectors. For tracking the position of animals, two small LEDs mounted to the cap (front and rear) were recorded by a digital camera (1080p-30f/s). Video tracking was analyzed in real-time by LabVIEW software (National Instruments).

### Behavior control, data acquisition, and analysis

The behavior hardware (motorized doors, digital camera, sensors from the shutter) and laser power supply were connected to a computer board (National Instruments #USB-6341) and controlled by LabVIEW and MATLAB (MathWorks, Natick, MA). Neurophysiological signals were sampled continuously at 20 kHz on a 1024-channel digital acquisition system (http://www.hinstra.com/ready-solutions/dedas) (Hinstra Instruments, Szeged, Hungary). The broadband signals were high-pass filtered (0.8–5 kHz) offline for spike detection, and low pass filtered (0–300 Hz) and downsampled to 1250 Hz for local field potentials. For automatic spike sorting, we used Klusta, an open-source neurophysiological data analysis package in Python (Rossant et al. 2016). For manual refinement of sorting, Phy package (part of Klusta) was used.

#### Laser light stimulation of neurons

A blue laser (473 nm CrystaLaser CL-473-050—output power at the tip of optic fibers 5 mW ± 0.5%, measured by Thorlabs PM100D Optical Power and Energy Meter Console with S120C sensor controlled by analog input combined with a shutter) generated a 10 ms constant power stimulus train. The shutter was constructed from a disassembled hard drive, with the addition of a simple control circuit for the coil of the pivot arm and the spin of the disk. The shutter was built based on the concept of Maguire et al. (Maguire et al. 2004) but it was modified as follows: the spinning disk with a hole acted as the shutter (constantly spinning with a hole) which sequentially covered and uncovered the beam. The coil with the arm worked as a secondary “slow” shutter, which opened and closed the path of the output of the already-shuttered laser beam. In the off position of the arm, we could monitor the exact duration of the laser beam before we used it for stimulation, via a photodiode attached to the head of the arm. We used fiber optic rotary joint patch cables (Thorlabs #FT200EMT). Cholinergic neurons were optogenetically tagged by laser stimulation (10 ms train for 2 s, 5mW) at the end of each session (last five minutes of the session without any behavior). We tested each neuron with at least 100 stimuli. For each cell, we generated peri-light-stimulus time histograms (Fig. S3 third column: optostimulation) to analyze: the proportion of light-responsive neurons, defined as firing rate during light pulse > 2 SDs above or below control firing rate (2000 ms epoch before the stimulus).

#### Behavior-specific single-unit activity (Fig. 3)

**Fig. 3:**
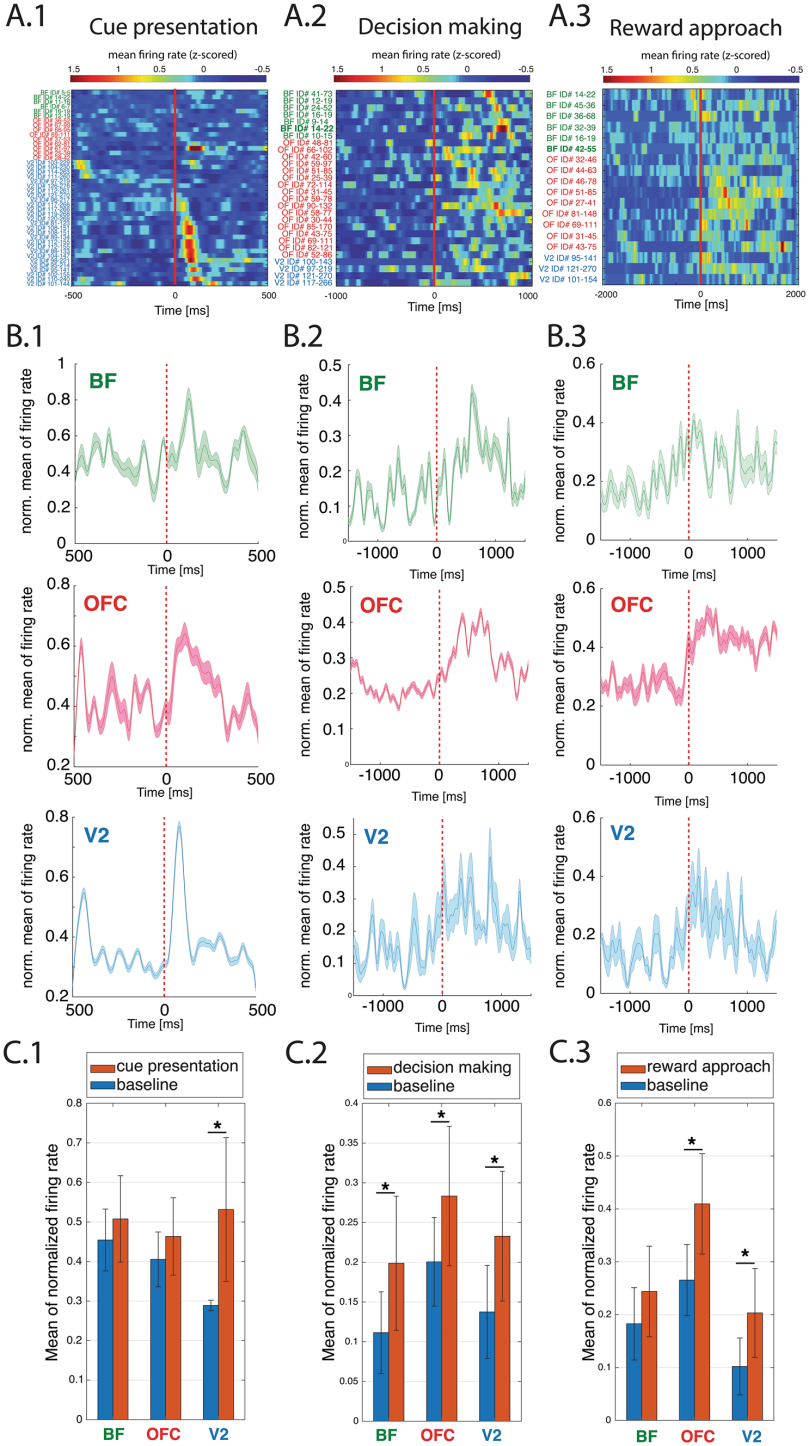
Firing modulation of BF, OFC and V2 cells during various behavior epochs (cue presentation, decision making, reward approach). The neurons were selected based on their firing pattern to the specific behavior from a pool of 147 V2 cells, 246 cells in OFC, and 137 cells in BF. **a.1** Individual Z-scores of responses of the BF (*n* = 6), OFC (*n* = 9), and V2 (*n* = 27) neurons during cue presentation. **b.1** The individual mean response of the same population of BF (green upper), OFC (red middle), and V2 (blue lower) neurons with + -STD. **c.1** The bars represent the comparison between the normalized firing rate of baseline and during cue presentation. Increased mean firing rate can be observed in V2 (Wilcoxon rank-sum test *p* < 0.001). **a.2 **Individual Z-scores of responses of the BF (*n* = 7), OFC (*n* = 17), and V2 (*n* = 4) neurons during decision making. **b.2** the individual mean response of the same population of BF (green upper), OFC (red middle), and V2 (blue lower) neurons with + -STD. **c.2** During the decision making, there was significantly increased neuronal activity in BF (Wilcoxon rank-sum test *p* < 0.001), OFC (Wilcoxon rank-sum test *p* = 0.0012), and V2 (Wilcoxon rank-sum test *p* = 0.0482). **a.3** Individual Z-scores of the response of BF (*n* = 6), OFC (*n* = 9), and V2 (*n* = 3) neurons during approach reward. **b.3** the individual mean response of the same population of BF (green upper), OFC (red middle), and V2 (blue lower) neurons with + -STD. **c.3** During approach reward, population activity of OFC (Wilcoxon rank-sum test *p* = 0.048), and V2 (Wilcoxon rank-sum test *p* = 0.0261) significantly increased. Stars above the bars mark the significance *p* < 0.05. Cell ID in green bold in (**A.2)** and (**A.3**) are cholinergic neurons

Changes in ongoing firing rates during different behavioral epochs were analyzed by comparing firing rates during cue onset, decision making, and reward approach epochs vs. baseline using Wilcoxon rank-sum test (Fig. 3, Fig S2). For baseline recordings, we used spike trains from the behavioral epochs when the rats were in the center of the maze at rest.

#### Coherence changes between behavioral epoch (Fig. 4)

**Fig. 4:**
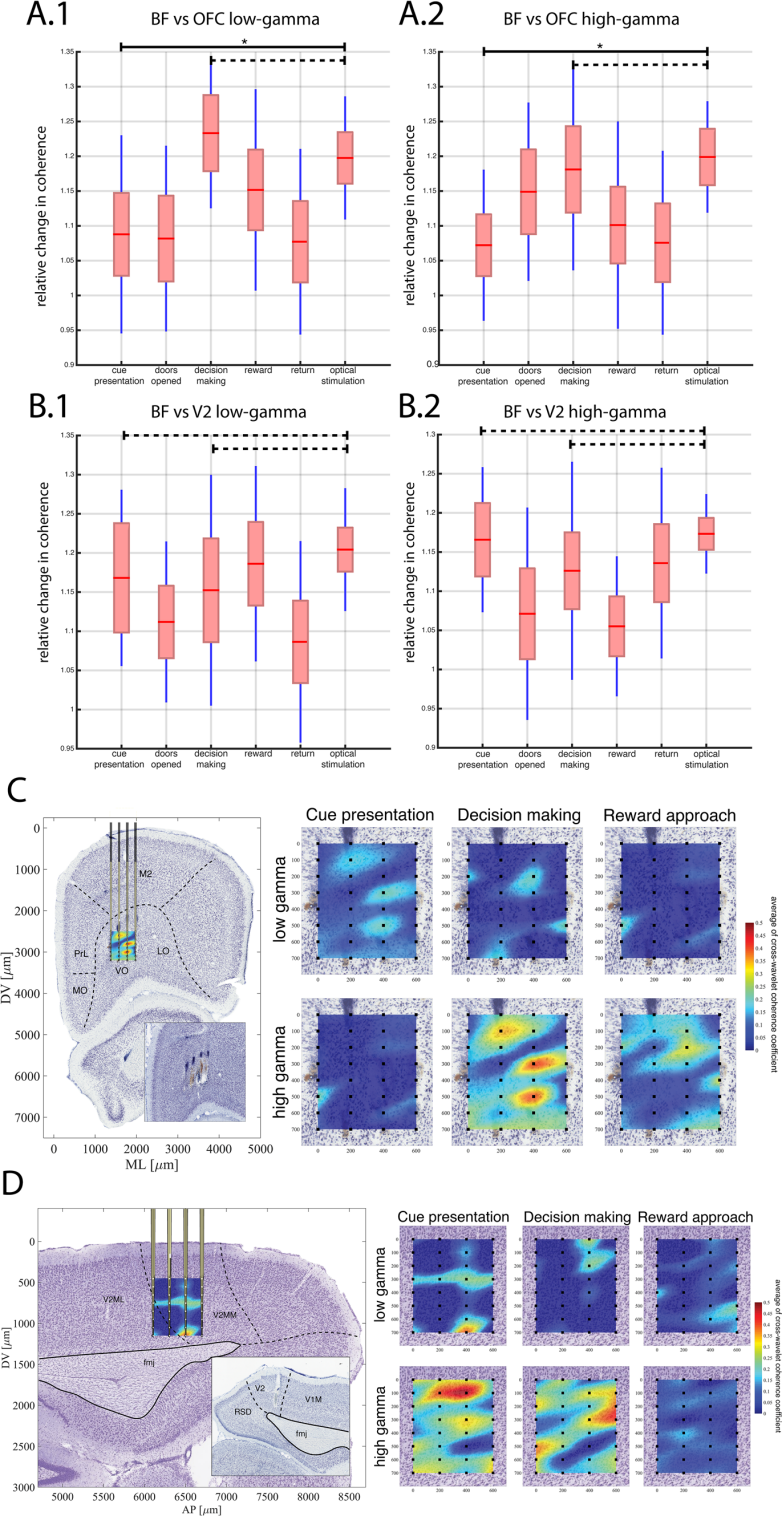
Mean of relative changes in coherence at low-low gamma band and in high gamma band between BF-OFC (**a.1**, **a.2**), and BF-V2 (**b.1**, **b.2**) during different behaviors and optical stimulation. Each bar indicates the median (the central red line), the 95% confidence intervals (box surrounding the red line) $\pm$ 1 SD. **a.1** Effect of behavior epoch and optic stimulation on relative coherence change between OFC vs BF at low-gamma band (*F*_(5,119)_ = 4.96, *p* = 0.0001). The relative change in coherence during cue presentation significantly differed from the optically stimulated values (Tukey’s HSD, *p* = 0.049 solid black line above bars). The decision-making condition did not significantly differ from the optically evoked coherence (Tukey’s HSD *p* = 0.96 dashed black line above bars). **a.2** There was a significant effect on the relative coherence change of high-gamma coherence (*F*_(5,131)_ = 4.13, *p* = 0.0016). Relative change in coherence during cue presentation was significantly different from the optically evoked values at the high-gamma band between OFC and BF (Tukey’s HSD *p* = 0.011 solid black lines above bars). However, during decision making, the relative change in coherence was not significantly different during the optical stimulation condition (Tukey’s HSD, *p* = 0.99 dashed black line above bars). **b.1** There was a significant effect on the relative change in coherence between BF-V2 at low gamma band (F_(5,116)_ = 3.57, *p* < 0.0049). However, coherence changes during cue presentation (Tukey’s HSD, *p* = 0.99) and decision making (Tukey’s HSD, *p* = 0.85) did not differ from the relative change in coherence during optical stimulation. **b.2** There was a significant effect on the relative coherence change between BF-V2 at high gamma (*F*_(5,116)_ = 3.47, *p* < 0.0058), but the cue presentation (Tukey’s HSD, *p* = 0.99) and decision making (Tukey’s HSD, *p* = 0.96) did not differ from the relative change in coherence during optic stimulation. **c** Coronal section showing orbital tracks of contact sites (black dots 4X8) of linear silicon electrode arrays, overlaid by spatially localized mean of cross-wavelet coherence at high gamma band between BF and OFC contact sites. Inset shows a coronal section with electrode tracks. The black dots on each surface plot represent the contact sites from OFC silicon electrode arrays. X–Y axes show the relative distance between contacts in µm. **d** Sagittal section showing V2 contact sites (black dots) of linear silicon electrode arrays, overlaid by spatially localized mean of cross-wavelet coherence at low gamma band between BF and V2. Inset shows a coronal section with the electrode tracks. The black dots on each surface plot represent the contact sites from V2 silicon electrode arrays. X–Y axes show the relative distance between contacts in µm

Using LFPs simultaneously recorded in multiple brain structures, we calculated the coherence between the structures in the low and high gamma bands. Continuous Wavelet Transformation (CWT) was used to obtain a time–frequency spectrogram of LFPs on all channels from BF, OFC, and V2 to assess behaviorally-related changes (Fig. S1 a, b, c). The coherence values of behavioral epochs and during optic stimulations were normalized as a proportion of baseline coherence, which was measured at the beginning of each trial when the animals were located at the center start position of the maze, with the doors closed. Significant changes in coherence (averaged across all channel pairings) were detected via one-way ANOVA followed by multiple comparisons between the relative coherence changes using Tukey’s Honestly Significant Difference Procedure (Tukey’s HSD) to determine if the significant changes in coherence occurred in specific behavioral epoch types or during the post-session optical stimulation period (Fig. 4a, b). *p* value was corrected using Bonferroni correction to counteract the problem of multiple comparisons.

#### Spatiotemporal coherence changes between all contact sites (basalo-cortical, cortico-cortical) using Monte Carlo permutation test (Fig. 5)

**Fig. 5:**
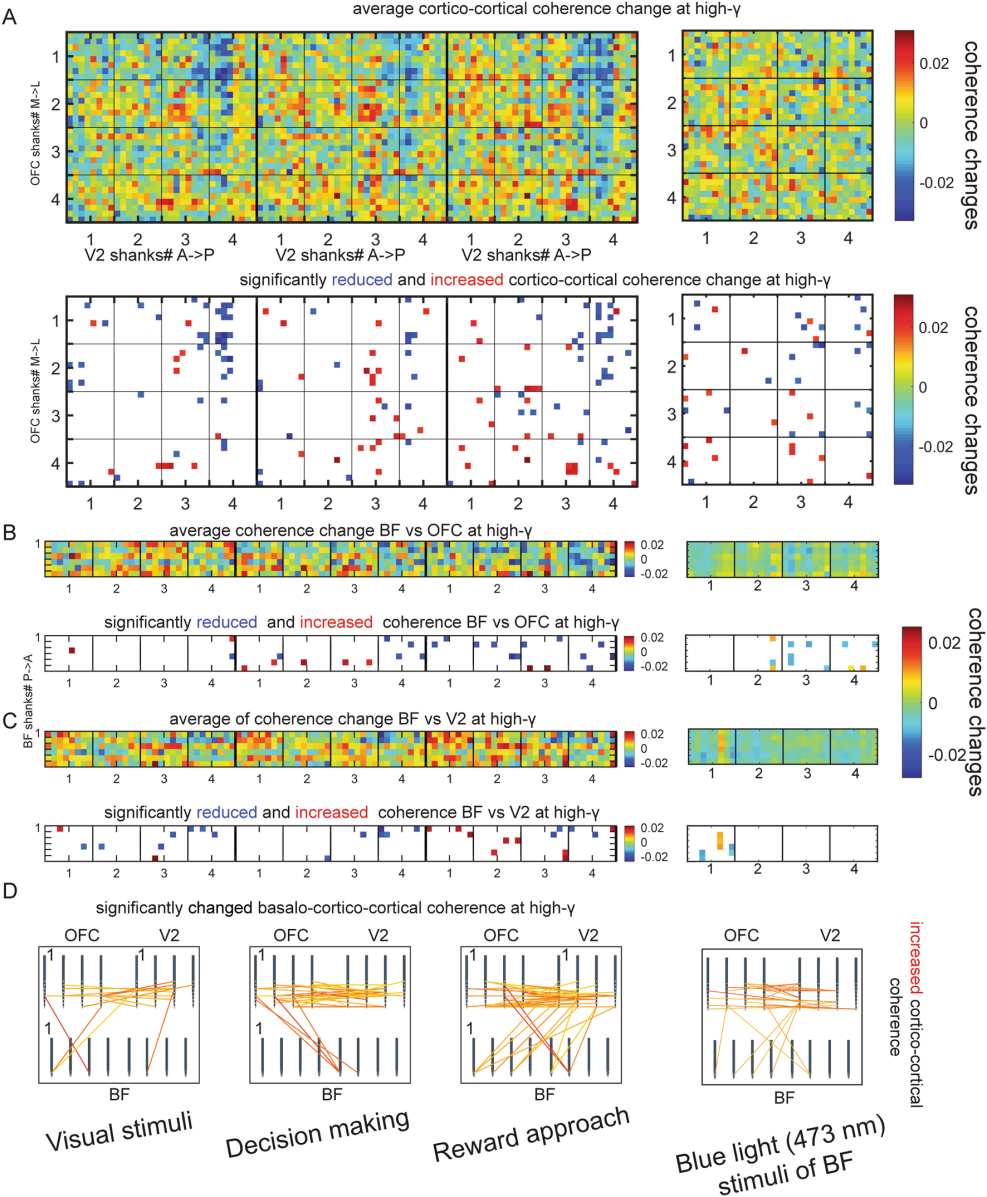
Maps of basalo-cortical and cortico-cortical high gamma band coherence changes during behavior. The three columns of each subplot divided by thick lines represent the behavioral epochs left to right: cue presentation, decision making, reward approach. Each 32 × 32 matrix represents a coherence map between electrode contacts on shanks (shanks#: *x*-axis V2, y-axis OFC). **a** The upper plot is the mean of cortico-cortical coherence change; the lower plot is the significant coherence change map. **b** upper plot: BF vs OFC coherence change; lower plot significantly changed coherence map, **c** upper plot: BF vs V2 coherence map; lower plot significantly changes coherence map. **d** Significantly increased spatiotemporal basalo-cortical and cotico-cortical-coherence maps using schematic arrangement of electrode locations representing contact pairs between OFC-V2-BF. In each box, lower 8 lines BF, upper left lines 4 OFC and upper right 4 V2 shanks. The fourth column represents data during blue light stimulation of BF using the same illustration method as during behavior in the left three columns. Color bars to the right indicate the extent of changes

For comparison across structures, continuous wavelet coherence (CWC) was calculated between each BF LFP recording site and each cortical recording site (basalo-cortical coherence), and between each V2 site and each OFC contact site (cortico-cortical). Confidence intervals for the event-related cross-coherence were calculated in space and time by Monte Carlo permutation tests or random permutation tests. First, we made a merged distribution of all CWC of LFPs between all combinations of channels from resting “center’ behavior epoch (2000 trials) combined with all trials from the event-related coherence. Next, we randomly sampled trials of CWC of LFPs from the combined distribution and then we calculated the mean coherence of both populations, and then calculated the difference score between the two means of coherence This random sampling procedure was repeated 3000 times to create a distribution of statistic values. Finally, we compared the observed coherence change score to the distribution that was generated by the result of the bootstrapped values. If the observed difference was greater than the 95% of the bootstrapped values, we rejected the null hypothesis that the two behavioral conditions resulted in the same changes in coherence between the given combination of channels (*n*).

$${\text{Coherence change score}}_{n}=\frac{{\text{mean}}\left({\text{CWC of BEHAVIOR}}\right)-{\text{mean}}({\text{CWC of RESTING}})}{{\text{mean}}\left({\text{CWC of BEHAVIOR}}\right)+{\text{mean}}{\text{(CWC\, of\, RESTING)}}}$$

The coherence change scores were used for constructing a functional spatial map between BF and cortical locations. The analysis was also repeated between the resting and optically stimulated time periods.

#### Spike-triggered averaged LFP (Fig. 6)

**Fig. 6:**
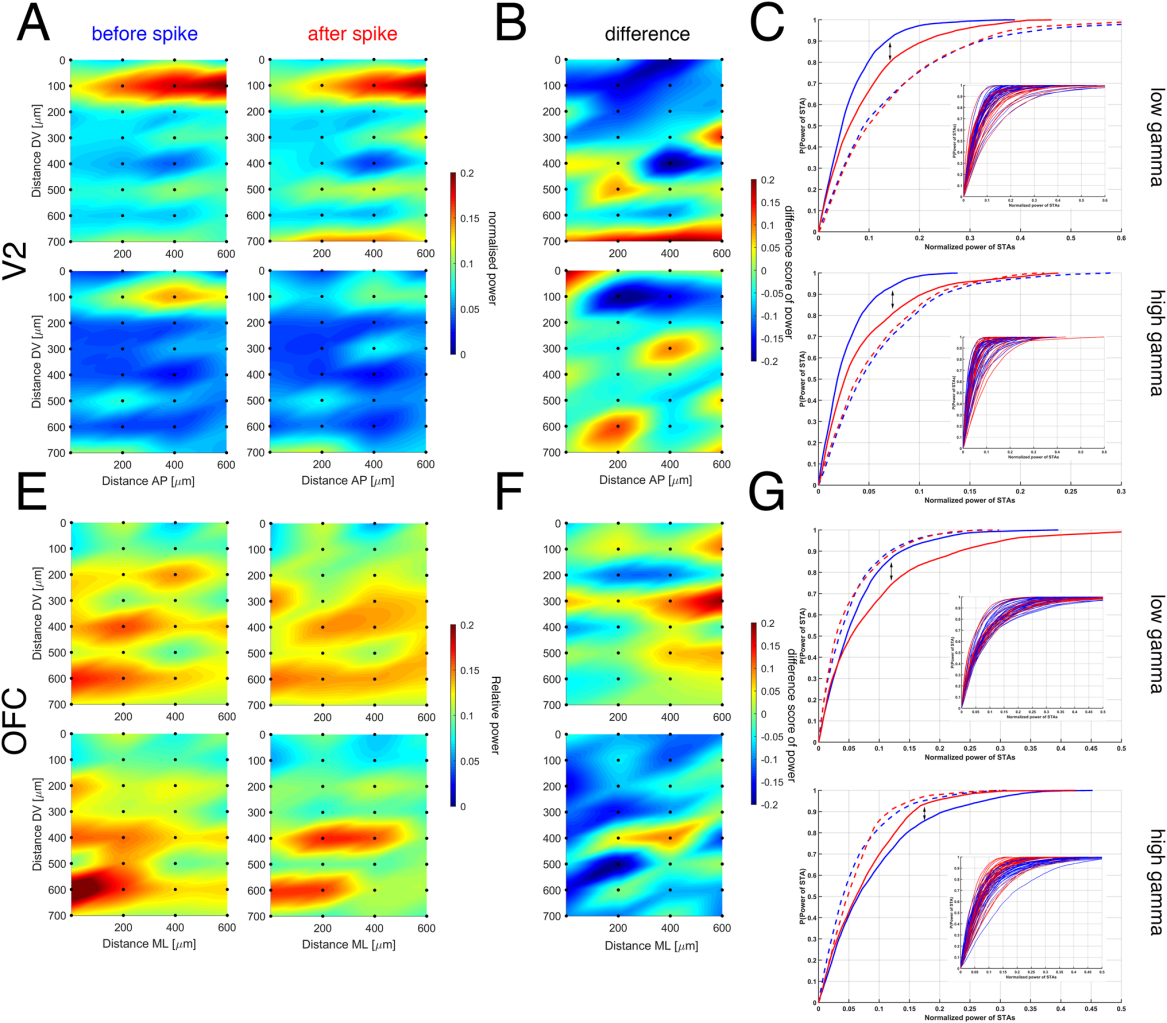
Plots of spike-triggered averages (STA) of the local field potentials from V2 (**a**), and from OFC (**e**) at specific cortical electrode sites (black dots) triggered by cholinergic cells (BF ID 42-55). The first row of **a** shows the power of STA at low gamma band from V2 and the first row of **e** corresponds with the low gamma power of STA from OFC, in contrast, the second row of **a** and **e** shows high gamma activity for given structures. “Before” above the first column with (blue) and “after” above the second column (red) represent the spatial gamma power activity before the spike and after the spike. The extracted LFPs time windows were 500–500 ms. Panels **b** and **f** show the difference score of STAs at low, and high gamma. **c** V2 and **g** OFC show the exponential cumulative distribution of low and high gamma power triggered by cholinergic spikes before the spike (blue) and after (red) lines. The continuous lines show the significant (*p* < 0.01) difference between cumulative distribution functions of the exponential distributions of the power before and after the cholinergic spike at a specific location. In contrast, the dashed lines show a non-significant differences between the distributions. The subplots show the distributions for each of the channels before (blue) and after the cholinergic spikes (red) lines at low gamma (first row) and high gamma frequency (second row) bands from V2 (**c**) and OFC (**g**). Dorso-ventral (DV) and antero-posterior (AP) location of contact sites are indicated on the Y and X

The oscillatory pattern of cortical areas modulated by cholinergic input was calculated by averaging cortical LFPs, triggered by BF cholinergic spiking. We calculated the empirical cumulative distribution function of the collected power value in low-gamma and high-gamma bands from the continuous wavelet-transformation of the cortical LFPs ± 500 ms around cholinergic spikes. A two-sample Kolmogorov–Smirnov test was applied to compare the empirical cumulative distribution of gamma power before and after cholinergic spikes to reveal significant differences (Fig. 6c, g).

#### Detection of short timescale interactions of neuron pairs and physiological features (Fig. S4 a, b)

Cross-correlograms of spike trains of neuron pairs can reveal putative synaptic connections between them (Barthó et al. 2004; Fujisawa et al. 2008; Stark and Abeles 2009). This takes the form of short time-lag (1–6 ms) peaks/troughs with positive or negative deviations from baseline, indicating putative excitatory or inhibitory connections, respectively. Such detection is based on testing the null hypothesis of a homogeneous baseline at a short time-scale (Stark and Abeles 2009). To this end, cross-correlograms binned in 0.5-ms windows were convolved with a 10-ms standard deviation Gaussian window resulting in a predictor of the baseline rate. At each time bin, the 99.99th percentile of the cumulative Poisson distribution (at the predicted rate) was used as the statistical threshold for significant detection of outliers from baseline. A putative connection was considered significant when at least two consecutive bins in the cross-correlograms within + 1.5 to + 5 ms passed the statistical threshold (Senzai and Buzsáki 2017).

#### Unit classification based on physiological features (Fig. S4c–i)

Cholinergic units were classified based on the trough-to-peak latency (TP latency) and the first two principal components of the second derivative of their waveform using the k-means clustering method. To calculate the TP latency, the averaged waveforms were taken from those recording sites where the amplitude shows the maximum deviation. The same averaged waveforms were used to perform PCA using the time period between 0 and 0.8 ms from the second derivative of the up-sampled averaged waveform. As a result of the waveform PCA, w-PCA1 and w-PCA2 were obtained. Burst index was determined by calculating the average number of spikes in the 3–5 ms bins of the spike autocorrelogram divided by the average number of spikes in the 200–300 ms bins. For k-means clustering, only the TP latency, w-PCA1, and w-PCA2 features were used. Group assignments resulting from k-means were used for supervised learning methods as labeled data. In order to cross-validate the k-means clustering, the TP latency, w-PCA1, w-PCA2 and mean firing rate were all used as features to train the supervised decision tree model (Bootstrap Aggregation “bagging” of Decision Trees model; MATLAB—Learner App., MathWorks, Natick, MA). The cholinergic cells were not part of the training set. With this method, we ensured that our k-means classified data is well-separable. Later, the model was used to predict the k-means group labels for identified cholinergic cells.

#### Statistical tests used

In Fig. 4a1-2 b1-2, ANOVA and Tukey's HSD test were used to reveal whether specific behavioral epochs and optical stimulation are linked to changes in BF-cortical coherence versus baseline. For Fig. 5, Monte Carlo permutation tests or random permutation tests for each pairing of recording sites helped us reveal significant anatomical location-dependent coherence differences that depended on the behavior or on optic stimulation of BF. The Kolmogorov–Smirnov (KS) test was used to compare gamma-band power values within the cortex before and after cholinergic spikes recorded from BF (Fig. 6). The main question was whether or not the distribution of gamma power at certain locations in the cortex in the period before 500 ms and after 500 ms of the cholinergic spike is the same or not? Did it change or not? If KS test shows significant differences, the power of gamma values comes from a different distribution (Fig. 6c, g). Thus, the firing of a cholinergic cell modulated the gamma power at specific locations in the cortex, but not globally.

## Results

### Anatomy of cholinergic projections to OFC and V2 (Fig. 2)

To demonstrate the cholinergic projection from the basal forebrain to OFC and V2, we used retrograde viral tracing. Although cholinergic neurons projecting to V2 are located throughout the whole extent of the diagonal band of Broca/substantia innominata (Huppé-Gourgues et al. 2018), OFC-projecting cholinergic cells tend to concentrate more caudal in the BF, including the internal capsule and adjacent globus pallidus region (Gielow and Zaborszky 2017). In one case, using monosynaptic virus-tracing, cholinergic neurons that collateralized to both OFC and V2 were found from − 0.4 to − 2.8 mm to the bregma (Paxinos and Watson 2007) in the internal capsule and adjacent globus pallidus (*n* = 13). The distribution of the various types of labeled neurons from this case are summarized in Supplementary Table S1. A comparison between cholinergic-specific innervation of OFC and V2 and the location of labeled cells in the BF from various experimental cases from the Allen Brain Institute (Allen 2011) also suggests that these two cortical areas receive joint projections from a limited BF area (see Fig. 2).

### Physiological properties of cholinergic and noncholinergic cells from BF, OFC and V2 (Figs. S2-S4)

We categorized recorded cells using a combination of techniques: a quantitative classifier based on the waveform and spiking cross-correlations to identify putative excitatory and inhibitory cells in the BF and in OFC and V2, and optogenetic tagging for identification of cholinergic cells in the BF (Fig. S4). First, the short-latency temporal interaction between all neuron pairs, using their spiking cross-correlation, identified excitatory and inhibitory units (Barthó et al. 2004) based on short (< 5 ms) latency offsets between two recorded neurons. The putative inhibitory and excitatory cells were grouped by trough-to-peak latency (TP-latency) using filtered waveforms (Fig. S4a. inset) and burst index (Fig. S4a). Combining these features (putative excitation and inhibition) with TP-latency resulted in three major groups of cells: excitatory cells (BF *n* = 19/137; OFC *n* = 159/246; V2 *n* = 109/147), narrow-waveform inhibitory cells (BF *n* = 89/137; OFC *n* = 28/246; V2 *n* = 7/147), and wide-waveform inhibitory cells (BF *n* = 0/137; OFC *n* = 18/246; V2 *n* = 11/147). The mean firing rates of excitatory neurons in BF (median = 5.8304 Hz), in OFC (median = 3.935 Hz) and in V2 (median = 5.275 Hz) were not significantly different (*p* > 0.05). The average firing rate of cholinergic neurons was 2.16 Hz and their average TP-latency was 0.6062 ms. Overall, the waveforms of cholinergic cells fell in the wide-waveform excitatory group, non-cholinergic neurons showed great heterogeneity and were classified in six different groups using K-means clustering and a supervised ‘bootstrap aggregating’ machine learning meta-algorithm (Fig. S4). Spiking activity of individual neurons from BF, OFC, and V2 are shown in Figs. S2, and Fig. S3.

### Cell responses during cue presentation, decision making and reward approach (Fig. 3)

To characterize the firing modulation of neurons in BF, OFC, and V2, we examined neuronal population responses during different behavioral epochs. During the task, animals were required to recognize and allocate attention to detect the frequency of visual stimulus presented on the doors of the center of the radial arm maze (Fig. 1b). After the doors opened for both directions, the animal decided to use one of the arms to approach the reward.

#### Visual cortex

During the cue presentation, the firing rate in some V2 cells changed relative to the condition when the animal remained in the center without cues (27/147; Wilcoxon rank-sum test; *p* < 0.05 criterion; Supplementary Fig. S2c, representative PSTHs in Supplementary Fig. S3d, e). More specifically, two cells showed ON–OFF responses (Fig. 3a1 ID#103-229; 104-230), two cells increased their firing rate during the offset of the stimuli (Fig. 3a.1 ID#114-263; 111-260), and the rest of the 23 neurons in V2 showed increased firing rate to stimulus onset (Fig. 3a.1). Before stimulus onset (baseline) the normalized (feature scaling) average firing rate of the V2 population was 0.2889, +-STD = 0.0132, in contrast, during the visual cue (Fig. 3c.1), the normalized average firing rate was 0.5314; +-STD 0.187. The population activity significantly increased during cue presentation (Wilcoxon rank-sum test *p* < 0.001). The population activity also significantly increased during decision making (*n* = 4 Fig. 3c.2, baseline normalized mean of firing rate: 0.1450 + -STD: 0.0728, during decision making the normalized mean of firing rate: 0.2492 + -STD: 0.0926; Wilcoxon rank-sum test *p* = 0.0482) and reward approach (*n* = 3 Fig. 3c.3 baseline normalized average population firing rate of neurons: 0.1384, during reward approach: 0.2229, Wilcoxon rank-sum test *p* = 0.0261).

#### Orbitofrontal cortex

In the OFC, we encountered cells that were modulated during cue presentation (Fig. 3a.1) but not significantly at the population level (9/246 norm. average of firing rate before cue presentation (baseline): 0.4053 + -STD: 0.0693, 0.4633 + -STD: 0.0979 during visual cue); however, cells significantly increased firing during decision making (Fig. 3c.2: 17 cells out of 246; baseline average normalized population firing rate before decision making: 0.2122 + -STD: 0.0384; during decision making: 0.2993 + -STD: 0.07; Wilcoxon rank-sum test *p* = 0.0012), and also increased significantly during reward approach (9/246 Fig. 3c.3 baseline normalized average firing rate before reward approach: 0.2798 + -STD: 0.0527 and during reward approach: 0.4403 + -STD: 0.0523, Wilcoxon rank-sum test *p* = 0.048).

#### Basal forebrain

BF cells showed heterogeneous responses. In the spike train of the BF (Supplementary Fig. S1f), two types of activity can be observed: cells with high firing rates and those with very low firing rates (< 1 Hz). Those cells showing low firing rates were identified as cholinergic based on optogenetic tagging. One cholinergic neuron significantly increased its firing during the reward approach (1/137; BF ID#42-55 in Fig. 3a.3 and in Supplementary Fig. S2 #55). Another cholinergic neuron showed increased firing during decision making (BF ID#14-22 Fig. 3a.2, Supplementary Fig. S3a #14-22). We found a few non-cholinergic neurons that were modulated by cue presentation, but their normalized averaged firing rate did not change significantly (6/137, Fig. 3c.1). During decision making (7/137; Fig. 3a.2, b.2) their normalized average firing rate together with cholinergic neurons #14-22 was 0.1170 + -STD: 0.0638 before decision making (baseline), which significantly increased to 0.2101 + -STD:0.0914 during decision making (Fig. 3c.2 Wilcoxon rank-sum test *p* < 0.001). Together with other BF neurons (6/137), the averaged normalized firing rates were 0.1938 + -STD: 0.0729 before the reward approach and 0.2567 + -STD: 0.0884 during the reward approach: (Fig. 3B.3, C.3).

### BF-OFC and BF-V2 relative coherence changes are similar between decision making and during optical stimulation (Fig. 4)

Oscillations in neuronal circuits can spatiotemporally coordinate the transmission of information. To address the temporal dynamics of oscillatory interactions between BF and cortex, basalo-cortical coherence changes were calculated using LFPs from the BF, OFC and V2 (see methods). To decrease the complexity of the investigations, we focused on the low and high gamma bands for further analysis. Supplementary Fig. S1a, b, and c show representative continuous wavelet transformation (CWT) of LFPs from the three regions during a single trial between 1 and 110 Hz. To address the variability between animals and between trials within a rat, the coherence values of behavioral epochs and during optic stimulations were normalized as a proportion of baseline. Baseline coherence values were measured when the animals were resting, positioned in the center of the maze, with closed doors for each trials. We concatenate the values of the proportion of coherence of each trials, from the forty-five sessions (averaged trial number: 15/session SD- + 2.3) of the three animals.

#### BF-OFC relative coherence changes

The relative BF-OFC coherence changes in both the high and low gamma ranges were elevated during the decision-making behavioral epoch of the task (Fig. 4a.1-a.2). To compare changes in coherence across each behavioral epoch and optical stimulation, a one-way ANOVA was conducted. We found that there were significant coherence changes within the low gamma band for the six conditions (Fig. 4a.1; *F*_(5,119)_ = 4.96, *p* = 0.0001). Next, we performed multiple comparisons (Supplementary Table 2 a–b) between the relative coherence changes using Tukey’s Honestly Significant Difference Procedure to determine if the significant changes in coherence occurred between particular behavioral epochs (cue presentation, decision making, etc.). Post hoc comparisons indicated that the relative coherence change of the BF-OFC low-gamma band during optical stimulation significantly differed from the coherence change during cue presentation (optical stimulation $\overline{x }=1.197 \pm 0.027$; cue presentation $\overline{x }$=1.087 $\pm$ 0.027, *p* = 0.049) but not during decision making ($\overline{x }$=1.2332 $\pm$ 0.033, *p* = 0.96; Fig. 4A.1; solid and hyphenated lines above the individual bars). There was also a significant effect on the relative coherence change of high-gamma coherence between BF and OFC (*F*_(5,131)_ = 4.13, *p* = 0.0016; Fig. 4a.2). Post-hoc comparisons revealed that the relative coherence change during the optical stimulation differed significantly from the coherence change during the cue presentation epoch (cue presentation $\overline{x }=1.072 \pm 0.026$; optical stimulation $\overline{x }=1.189 \pm 0.024$; *p* = 0.011). However, similar to the low gamma band comparisons, the relative coherence change during decision making did not differ significantly from the optically evoked coherence change at the high gamma band (decision making $\overline{x }=1.181 \pm 0.027$; *p* = 0.99; Fig. 4a.2). Taken together, these results suggest that optically evoked changes in relative coherence between BF and OFC were similar to the relative change in coherence during decision making, and they differed from the change in relative coherence during cue presentation.

#### BF-V2 relative coherence changes

There was a significant effect on the relative coherence change between BF-V2 at low gamma band for the five behavioral epochs and optical stimulation (Fig. 4b.1; *F*_(5,116)_ = 3.57, *p* < 0.0049). However, the post hoc analysis (Supplementary Table 3 a-b) indicated that the relative change of the optically evoked coherence ($\overline{x }=$ 1.204 $\pm$ 0.0276) did not differ from the relative change in coherence during cue presentation (optical stimulation $\overline{x }=1.204 \pm 0.0276$; cue presentation $\overline{x }=1.168 \pm 0.028$; *p* = 0.99) or during decision making ($\overline{x }=1.152 \pm 0.021$; *p* = 0.85). Similarly, at the high gamma band, there was a significant difference between the relative change in coherence (Fig. 4b.2; *F*_(5,116)_ = 3.47, *p* < 0.0058). However, the visual cue ($\overline{x }=1.165 \pm 0.029$; *p* = *0.99*) and decision making ($\overline{x }=1.126 \pm 0.02$; *p* = 0.96) behavioral epochs did not significantly differ from the optically evoked change in coherence ($\overline{x }=1.153 \pm 0.026$).

The above statistical analysis revealed similarities between specific behavioral epochs and optic stimulation. Beyond the temporal dynamic changes in coherence during the behavioral epochs, we aimed to reveal whether the coherence displays homogeneous or spatially focal maxima between the BF and the cortex. Panels in Fig. 4c–d demonstrate behavior-dependent spatially localized coherence events; we calculated averaged wavelet coherences between a single BF contact site (mean of LFPs of contact sites of 5th shank) vs the OFC at each contact site (individual LFPs of all cortical contact sites). Figure 4c demonstrates the spatio-temporal “co-oscillation channels” around localized contact sites in the OFC within each behavior epoch, including decision making and reward approach at low and high-gamma bands. The coherence at the high-gamma band seems to be more robust spatially than during cue presentation or reward approach.

The average BF-V2 coherence has spatially distinct local maxima around specific array contact sites (Fig. 4d). Basalo-cortical coherence increased in the low-gamma band during cue presentation, and a horizontal coherence “layer” can be observed around 300 µm depth. This horizontal-shaped coherence disappeared during the decision-making and reward approach epochs in the low gamma (Fig. 4d, upper row). In the high gamma during cue presentation and decision making, several increased coherence patches can be observed. These results drove us to extend the analysis to investigate spatio-temporal coherence between BF and V2, OFC at all contact sites, as described in the next paragraph.

### Cortico-cortical coherence during behavior and cholinergic stimulation (Fig. 5)

We further analyzed detailed spatial coherence changes between all contact sites in the cortex and each shank in the BF using Monte Carlo permutation tests (Fig. 5). As the previous results revealed, the basalo-cortical coherence was spatiotemporally modulated during decision making simultaneously between BF/OFC and BF/V2 at high gamma band (Fig. 5, panels c and d); therefore, we tested cortico-cortical coherence changes during behavioral epochs. Each of the coherence change values were extracted between all combinations of contact sites, creating a coherence matrix consisting of 1024 voxels. These voxels represent LFP/LFP coherence changes in the three different behavioral epochs (visual stimulation, decision making and approach reward: Fig. 5a, first row, left matrix) and optic stimulation of BF (Fig. 5a, first row, right matrix). Each combination of contact sites indicates coherence change score; e.g., change between center versus cue presentation, decision making, or approach reward approach. Scores range from − 1 (decrease of coherence during behavioral epochs) to + 1 (increase of coherence during behavioral epoch). In Fig. 5 panel ‘a’ depicts the average cortico-cortical coherence change matrix during cue presentation, decision making and reward approach epochs between OFC and V2. To distinguish behavioral or optic stimulation-related coherence score changes, we calculated the critical values for each combination of coherence changes to determine the significance level (1% significance level) using a bootstrapping method (see methods). Figure 5 panel ‘a’, second row displays only significantly (alpha = 0.01) reduced (blue) or increased (red) locations of cortico-cortical coherence change at high gamma. Panels in Fig. 5b display average (first row) and significant coherence change (second row) pairs between BF and OFC. Finally, panels in Fig. 5c display average (first row) and significant coherence change (second row) pairs between BF and V2. The bottom row in Fig. 5 (panel d) shows schematically the significant spatio-temporal relations of functional connectivity between BF/OFC and BF/V2 in the distinct behavioral epochs at high-gamma band with illustration of individual shanks.

It is apparent that the number of significant coherence combinations is different in the specific behaviour epochs. The inhomogeneous spatial distribution of the coherence between the contact sites from the two cortical regions or between BF and cortical sites seems to depend on the behavioral epochs and the anatomical location of probes.

To compare the fine spatial distribution of basalo-cortical and cortico-cortical coherence changes during cholinergic stimulation, the right column of Fig. 5 uses the same type of illustration. The coherence differences suggest that the cholinergic system can modulate cortico-cortical gamma-related coherence patterns, enabling information processing between these cortical regions.

### Cholinergic spike-triggered averages of spatial cortical LFP (Fig. 6)

Spatial spike-triggered averages (STA) of cortical LFPs induced by cholinergic firing demonstrate their effect on cortical gamma power (Fig. 6). We calculated the power of low gamma and high gamma at every contact site in V2 (Fig. 6a) and in OFC (Fig. 6e) before and after the cholinergic spikes within a [− 500, 500 ms] time window. The difference scores showed power changes around spike events, which demonstrated differences of the power in low and high gamma bands in V2 (Fig. 6b), and OFC (Fig. 6f). In V2, the power of low gamma increased significantly around 300 µm, 500 µm and 700 µm relative depth within the cortex (Fig. 6b). Similarly, Fig. 6f for OFC shows increased spike-triggered averaged (STA) LFP patches at low and high gamma band.

The empirical cumulative distribution function (ECDF) represents the accumulated power values within a 1000 ms time window centred on each spike (Fig. 6c for V2 and Fig. 6g for OFC). A two-sample Kolmogorov–Smirnov test showed significant differences (*p* < 0.05) between the ECDF of before events, and that of after events across all cortical contact sites (Fig. 6c, g and Table 1). Table 1 summarizes the percentage of significant gamma changes at specific contact sites for all cholinergic cells, where the ECDF showed significant differences. For example, in the case of cholinergic cell ID16-39, one site showed significant differences in V2 (low gamma; Kolmogorov–Smirnov test; *p* = 0.016, ks2stat = 0.045) and the same cell displayed significant differences at another site in both low and high gamma, respectively in OFC (*p* = 0.041; ks2tat = 0.041 and *p* = 0.016, ks2stat = 0.046). Another cholinergic-induced STA analysis (cell ID14-22) shows significant differences at two sites in the OFC at low gamma (*p* = 6.118*10^−6^; ks2stat = 0.104 and *p* = 0.003, ks2stat = 0.075) and at high gamma: *p* = 4.83*10^−15^, ks2stat = 0.169 and *p* = 0.015, ks2stat = 0.064).

**Table 1:** Percentage of significant gamma changes at specific contact sites

Cell ID	V2		OFC		Shank# (location)
	Low gamma (%)	High gamma (%)	Low gamma (%)	High gamma (%)	
14–22	0	0	6.25	6.25	5
16–39	3.125	0	3.125	3.125	5
32–49	0	0	0	0	7
42–55	3.125	0	9.375	0	7
43–56	12.5	0	0	0	7
45–61	6.25	3.125	0	0	3

## Discussion

In this study, we investigated the functional circuitry between specific locations in the BF, orbitofrontal (OFC), and visual cortex (V2) with high-density electrode arrays during a visual discrimination task, performed by transgenic ChAT-Cre rats. It is known from anatomical studies that V2 and OFC are reciprocally connected (Reep et al. 1996), and we have identified in a single case that a few cholinergic neurons in the internal capsule/globus pallidus/substantia innominata region collateralized to innervate both V2 and the orbitofrontal cortex.

In the behavioral task, both in low and high gamma, coherence changed according to the behavioral demands between BF/OFC and BF/V2, respectively (Fig. 4). Moreover, we found distinct differences in the fine spatial distributions of coherence values between basalo-cortical and cortico-cortical sites during specific behavioral epochs (Fig. 5). For example, Fig. 5d reveals a hotspot of coherence between a specific BF and V2 sites (BF 6th shank vs V2 shank 3th) or between a specific BF and OFC sites (BF 6th shank-OFC 3th shank) from the same location in a behavior-dependent manner. It only exists during the specific behavior (during visual stimuli and reward approach). But the BF-V2 coherence, for example, during reward shows more global changes in terms of localization in contrast to visual cue. The spatial coherence pattern maintains across trials, even across sessions in the same behavior epoch and anatomical localization. The spatial map of coherence, however, is not static, but dynamically changes between behavioral epochs. Moreover, laser stimulation of BF cholinergic cells generates spatial coherence changes among specific cortico-cortical sites (Fig. 5 right column), and cholinergic spike triggered averages of cortical LFPs showed significant differences before and after cholinergic spikes at specific cortical contact sites (Fig. 6, Table 1), suggesting that the cholinergic system contributes to selective modulation of cortico-cortical circuits. Across behaviors and task demands, coherence between disparate but connected brain regions dynamically changes under the control of specific BF sites. Mapping the cortical coherence changes together with the location of electrode arrays in the BF (Fig. 5d) suggests that neurons in specific sub-regions in the BF are dynamically recruited during the various behavioral epochs to coordinate specific cognitive processes.

### BF oscillatory dynamics and their correlation with discrete cortical oscillations

Recent large-scale recording of the BF shows that neurons fire with peaks in different frequency ranges that strongly correlate with specific behavioral epochs, including target detection, decision making, and outcome evaluation (Tingley et al. 2014, 2015, 2018). Although these authors seldom used simultaneous recording in the BF and related cortical areas, they suggested that these ‘nested’ oscillations in the BF (‘multiplexing’) might maximize the transfer of segregated information to remote cortical regions in a sequentially organized fashion. In our task, low and high gamma (40–100 Hz) dominated the three investigated brain regions in all behavioral epochs, except for a strong theta power in V2 (Supplementary Fig. S1).

There is a vast literature to suggest that neuronal interactions are modulated through synchronization (Womelsdorf et al. 2007). Gamma-band synchronization involves rhythmic inhibition of local cortical networks (Cardin et al. 2009; Kim et al. 2016); the periods between inhibition provide temporal windows for neuronal communication. If a stimulus is selected by attention, communicating cortical areas show stronger and higher frequency gamma-band synchronization (Lakatos et al. 2008; Gregoriou et al. 2009; Buschman and Kastner 2015; Fries 2015). Basal forebrain cholinergic input has been suggested to be involved in attentional modulation of cortical circuits (Hasselmo and McGaughy, 2004; Sarter et al., 2009; Schmitz and Duncan 2018; Thiele and Bellgrove 2018) but the cellular, circuit and synaptic mechanisms are complicated and in most cases remain to be elucidated.

Gamma band directional interactions were shown between BF and visual cortex during wake and sleep states (Nair et al. 2016) and BF stimulation enhances perception linked to LFP changes in visual cortical gamma (Goard and Dan 2009; Pinto et al. 2013). Furthermore, it has been shown that ACh release in the mPFC promotes gamma oscillations (Howe et al. 2017). Observations in the somatosensory cortex of mice, similarly, increased cholinergic input is linked to changes of high and low-frequency components of cortical LFPs (Eggermann et al. 2014; Kalmbach and Waters 2014).

We have shown that BF laser stimulation in ChAT-Cre rats induces significant coherence changes in high gamma in the OFC/V2 network at specific sites (Fig. 5, right column). BF neurons enhance gamma coherence between specific V2 and OFC sites that are likely to be interconnected. The fact that we did not observe significant coherence changes between all electrode pairs argues for the specificity of the main effect and suggests that it is not a methodological artifact.

There is good evidence that cortically projecting BF PV neurons are involved in cortical gamma band oscillations at 40 Hz (Yang et al. 2014; Kim et al. 2015; Dannenberg et al. 2015); however, this phenomenon may relate to BF cholinergic neurons exciting BF GABAergic projection neurons through cholinergic collaterals synapsing on PV cells (Zaborszky and Duque 2000).

### Cell assemblies in the BF

Previous research (Lin et al. 2006) observed that a subset of BF neurons, called tonic firing neurons, bursted as transient (160 ms) ensembles, and their synchronization phase was tightly associated with prefrontal oscillation power. The responses of these putative non-cholinergic neurons correlated with the motivationally salient sensory cues that reliably predicted reinforcement, associated motor responses, and hedonic valences (reward or punishment), irrespective of their sensory modalities. However, BF bursting was absent when the same sensory cues were not motivationally salient (Lin and Nicolelis 2008). Studies by Nitz, Chiba and their colleagues emphasized that BF neurons form distinct task-phase ensembles within theta, beta, low and high gamma frequency windows (Tingley et al. 2018).

Defining short-latency functional interactions using a spike-jittering cross-correlation on a small subset of BF neurons suggests that the temporal interaction between two functional assemblies may be as little as 10–15 ms (Zaborszky and Gombkoto 2018). Behavior-dependent synchronization of BF neurons into cell assemblies in response to environmental or internal demands could be a mechanism by which widely separated cortical regions are quickly coordinated to facilitate or inhibit information flow between them as needed. This notion was predicted already from our early anatomical studies suggesting that “the BF is well-positioned anatomically to coordinate cortical oscillations among widely separated cortical regions and capable of binding these regions into larger functional networks” (Zaborszky 2002; Zaborszky et al. 2002). While studies by Nitz, Chiba and their colleagues suggest temporal coordination of BF neurons to form neuronal ensembles, our studies, by showing subtle differences in basalo-cortical coherence sites as assessed from the location of electrodes in the BF (Fig. 5d), indicate that cell ensembles in topographically different BF locations might be dynamically recruited during the various behavioral epochs.

### Cholinergic network in behaving rodents

To our knowledge, cholinergic neurons in behaving rodents were identified first by Hangya et al. (Hangya et al. 2015) and Harrison et al. (Harrison et al. 2016). Cholinergic neurons responded to reward and punishment, similarly to unidentified BF neurons in primates by Monosov and his colleagues (Monosov et al. 2015; Zhang et al. 2019).

Our observations that the cholinergic neurons identified by optogenetic tagging (BF ID14-22, Fig. 3.a2) responded during decision-making or (BF ID 42-55; Fig. 3.a3) during reward-approach, and also that their STA modulated LFPs in specific OFC and V2 sites (Fig. 6, Table 1) suggest BF cholinergic modulation of specific cortical circuits. Nicotinic and muscarinic receptors are involved in specific modulation of gamma power in the prefrontal cortex (Kalmbach and Waters 2014). Due to the differential distribution of muscarinic and nicotinic receptors on pyramidal and various interneurons (Muñoz and Rudy 2014; Verhoog et al. 2016), the existence of two types of cholinergic cells in the BF of mice (Unal et al. 2012; Bloem et al. 2014), and the relative paucity of identified cholinergic neurons in our study, further research is necessary to find out which microcircuits are affected (Hasselmo and Cekic 1996; Xiang et al. 1998).

### Conjoint modulation of functional connectivity between visual and orbitofrontal cortex

Orbital areas have reciprocal connections with V2, as shown using classical anterograde and retrograde tracers (Vogt and Miller 1983; Miller and Vogt 1984; Sanderson et al. 1991; Paperna and Malach 1991; Van Eden et al. 1992; Reep et al. 1996). The OFC has been suggested to be involved in many functions, including response inhibition, flexible representation of stimulus-outcome association, value coding, prediction error, coding of reward probability, and emotional appraisal; implying that OFC neurons play a major role of valuation and decision making. Neurons in the OFC respond both to primary reinforcers, as well as cues that predict rewards across multiple sensory domains, including visual, gustatory, somatosensory, olfactory, and auditory (Rolls and Deco 2006; Schoenbaum et al. 2009; Mainen and Kepecs 2009; O’Neill and Schultz 2010; Burke and Tobler 2011; Passingham and Wise 2012; McGinty et al. 2016; Sharpe and Schoenbaum 2016; Wikenheiser and Schoenbaum 2016; Izquierdo 2017).

In the OFC, we detected neurons that responded differently to visual cues (OFC neuron #61-97, see Supplementary Fig. S3c) in the same region, where cells were encountered that responded while approaching the reward. In the OFC and V2 during decision making, and in V2 also during cue presentation, the gamma coherence increased; furthermore, the cholinergic STA in both cortical areas show spatially increased gamma power, thus it is likely that cholinergic neurons are involved in modulating gamma coherence between OFC/V2 regions during the decision to approach the reward.

## Concluding remarks

The electrophysiological data suggest that cholinergic neurons together with other BF neurons participate in spatio-temporal coordination of cortical activity. This coordination may entail the exchange of information between specific interconnected cortical regions, which might underlie particular aspects of cognitive functions. This suggestion is in line with experiments showing coordinated ACh release in PFC and hippocampus (Teles-Grilo Ruivo et al. 2017). Acetylcholine can rapidly modulate the activity of specific circuits in the cortex (Sarter and Lustig, 2020). Although we focused on BF coordination of cortical ACh release, other mechanisms, including cortico-cortical, thalamo-cortical, and intrinsic connections between specific BF neurons may also play a role.

These data and past anatomical studies (Li et al. 2017; Záborszky et al. 2018) suggest an emerging organization of the cholinergic system, in which functional cell assemblies may be dynamically recruited in correlation with behavior demands, through specific projections between distinct BF and cortical sites.

## Supplementary Information

Below is the link to the electronic supplementary material.

Supplementary file1 (TIF 47146 kb) Supplementary Fig. S1 Continuous wavelet transformation of a single trial of LFP signals from BF (**a**) OFC (**b**) and V2 (**c**). Vertical lines represent the behavioral epochs during the trial: visual cues were presented between the red lines. Purple line shows the time of opening door. Decision making was calculated as the epoch 2 sec before the white line (**a**,** b**,** c**) or black line (**d**,** e**,** f**,** g**) indicating the start of the approach to the reward. (**d**) shows the section of continuous wavelet transformation at low gamma (45-75Hz), and the (**e**) shows the section at the high gamma band. The green lines represent the LFP power from the BF at the specific frequency bands, the blue is from V2 and the red one is from OFC. (**f**) shows the single units during the trials, the color corresponds to the given structure: blue from the V2, red from OFC, and the green is from the BF. (**g**) shows the accelerometer row data at x-y-z axis in the space. Arrow represents the exact time point of the command to open the doors within the maze. Energy contents of the lower frequency bands (delta, alpha, beta) were low except for theta oscillations within the visual cortex. In the BF, the power of low-gamma oscillations (45-75Hz) were increased at the end of the decision making. Increased high-gamma oscillation (75-100) power can be observed around decision making and reward in BF and OFC. In the V2, low- and high-gamma bands are intense during the whole trial, but their occurrence density shows behavioral dependency

Supplementary file2 (TIF 16446 kb) Supplementary Fig. S2 Behavior-related changes in spiking activity of BF ChAT+ cells (**a**) and putative pyramidal/excitatory and interneuron/inhibitory neurons in OFC (**b**) and V2 (**c**) during different behavioral epoch across one session, 30 trials. The colored bars represent the behavioral epoch: blue – rat in center location representing the baseline, orange - cue presentation, yellow -decision making, purple – approach/reward. The responses were compared between baseline and other epochs by Wilcoxon rank-sum test; *p<0.05 were significantly different

Supplementary file3 (TIF 55483 kb) Supplementary Fig. S3 Neuronal responses of individual neurons from BF, OFC, V2. The first column is the firing maps in the maze [normalized firing rate/(occupancy time/bin)]. The second column contains some examples of responses during decision making (green background), approach reward (yellow background), and during visual stimuli (dark blue background). The third column shows PSTHs from 0 to the 2 sec illustrating the neuronal response to the blue laser stimulation (Optostimulation, blue background). The fourth column is the autocorrelogram of the cells with their waveforms across the contact sites of the silicon probe shank the cell was recorded from. (**a**) shows a cholinergic cell (BF ID# 14-22) response to decision making (green). (**b**) shows an OFC neuron that responded (yellow) to approach-reward. (**c**) is a “decision-making neuron” that responded differently to 5Hz vs. 1Hz visual cue. (**d**-**e**) two types of cells from V2, ON-OFF, and onset of visual cue (The red dashed line at 0 sec is the onset of the visual stimuli)

Supplementary file4 (TIF 12062 kb) Supplementary Fig. S4 Neural classification based on physiological features in BF, OFC and V2. (a) Single units (n=530 from 3 rats) were first preliminarily classified based on trough-to-peak (TP) latency and burst index. Wide waveform units were grouped into putative excitatory cells. Black line represents the distribution of putative excitatory cells (TP>0.485 or mean FR<6 Hz) and the dashed blue line shows the putative inhibitory cells (TP<0.485 or FR>6 Hz), subplot shows the trough-to-peak latency of mean waveform of a neuron. (**a**) Each dot corresponds to one unit from BF (green), OFC (red), V2 (blue) overlapped with putative excitatory (+) and inhibitory (o) markers. Right marginal distribution of burst index sorted by structures (BF green), (OFC red), (V2 blue). (**b**) examples of autocorrelogram and cross-correlogram of two putative excitatory cells and one inhibitory cell. First row from OFC (#73-87), second row from V2 (#2-#1) and third row from BF (#59-#54) with their waveforms. Scatter plot (**c**) green o- BF, (**d**) red o- OFC, (**e**) blue o- V2 superimposed with filled markers which correspond to the results of k-means clustering (six clusters) using the first (x-axis), and second (y-axis) W-PCA means second first and second PCA of second derivate waveform correspond to each of units (dots) with their TP latency (z-axis). (**f**) confusion matrix, x-axis corresponds to prediction results, y-axis shows the true class. (**g**) distribution of cells within k-means group with classified cholinergic cells marker (X), (**h**) mean of TC-latency grouped by k-means from BF, OFC and V2. (**i**) mean of firing rate grouped by k-means clustering. Each color represents the same cluster between (**f**), (**g**), (**h**), (**i**) subplots. Overall, the waveform of cholinergic cells falls into the wide-waveform excitatory group, and they are predictable based on their physiological features using the trained decision tree matching learning model. Thus, we can conclude that the recorded cells are heterogeneous in term of electrophysiological features, but the cholinergic cells show similar characteristics which led the prediction to classify them into a specific group

Supplementary file5 (XLSX 10 kb)

Supplementary file6 (DOCX 23 kb)

## Data Availability

All raw data are available from the authors.
