# Supplementary figures and images for: Contribution of the basal forebrain to corticocortical network interactions
Source: Brain Struct Funct. 2021 May 22;226(6):1803–21. doi: 10.1007/s00429-021-02290-z (PMC8203523; doi:10.1007/s00429-021-02290-z)

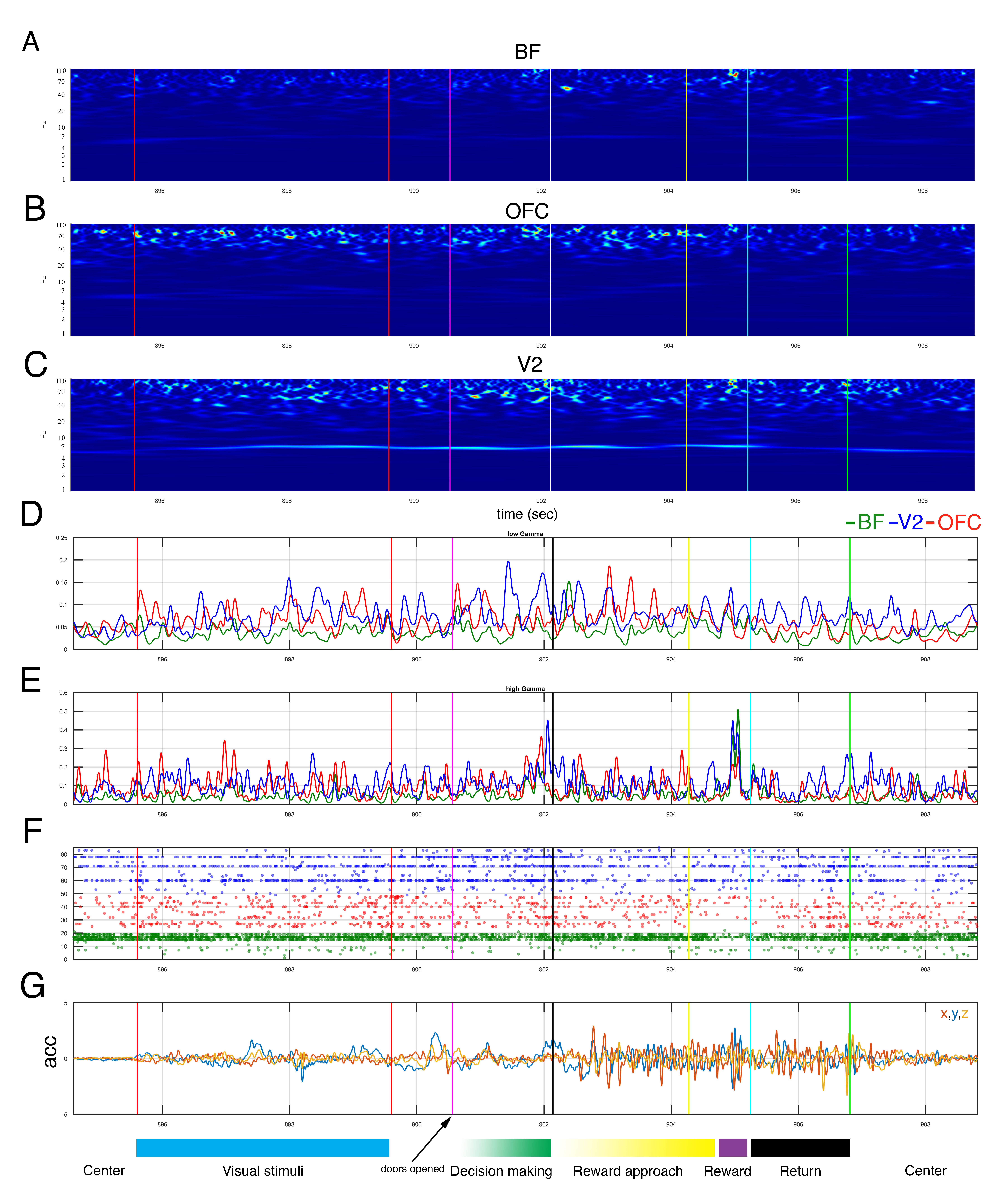

Supplement: Supplementary file 1 — Supplementary file1 (TIF 47146 kb) Supplementary Fig. S1 Continuous wavelet transformation of a single trial of LFP signals from BF (a) OFC (b) and V2 (c). Vertical lines represent the behavioral epochs during the trial: visual cues were presented between the red lines. Purple line shows the time of opening door. Decision making was calculated as the epoch 2 sec before the white line (a, b, c) or black line (d, e, f, g) indicating the start of the approach to the reward. (d) shows the section of continuous wavelet transformation at low gamma (45-75Hz), and the (e) shows the section at the high gamma band. The green lines represent the LFP power from the BF at the specific frequency bands, the blue is from V2 and the red one is from OFC. (f) shows the single units during the trials, the color corresponds to the given structure: blue from the V2, red from OFC, and the green is from the BF. (g) shows the accelerometer row data at x-y-z axis in the space. Arrow represents the exact time point of the command to open the doors within the maze. Energy contents of the lower frequency bands (delta, alpha, beta) were low except for theta oscillations within the visual cortex. In the BF, the power of low-gamma oscillations (45-75Hz) were increased at the end of the decision making. Increased high-gamma oscillation (75-100) power can be observed around decision making and reward in BF and OFC. In the V2, low- and high-gamma bands are intense during the whole trial, but their occurrence density shows behavioral dependency [file 429_2021_2290_MOESM1_ESM.tif]

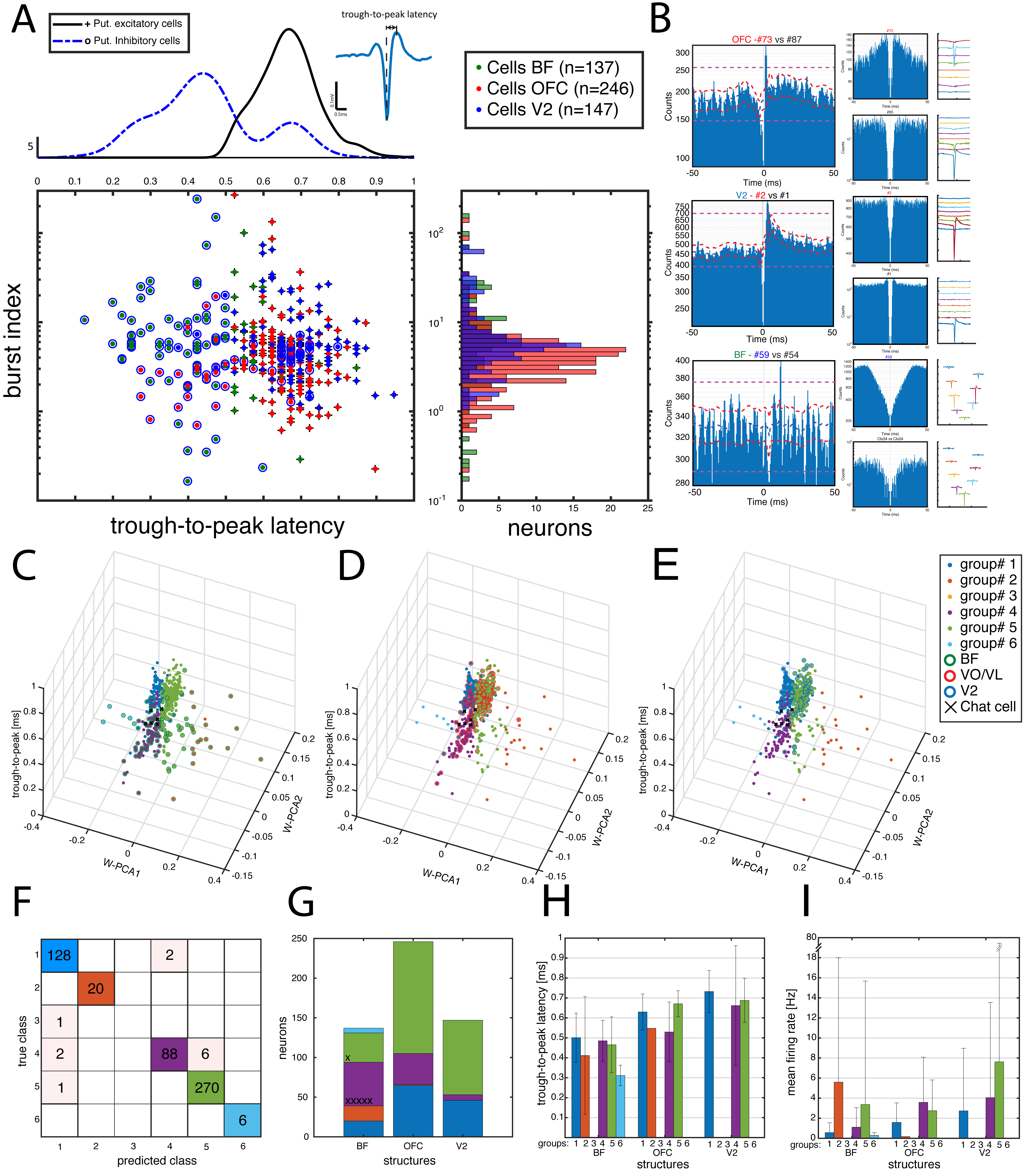

Supplement: Supplementary file 4 — Supplementary file4 (TIF 12062 kb) Supplementary Fig. S4 Neural classification based on physiological features in BF, OFC and V2. (a) Single units (n=530 from 3 rats) were first preliminarily classified based on trough-to-peak (TP) latency and burst index. Wide waveform units were grouped into putative excitatory cells. Black line represents the distribution of putative excitatory cells (TP>0.485 or mean FR<6 Hz) and the dashed blue line shows the putative inhibitory cells (TP<0.485 or FR>6 Hz), subplot shows the trough-to-peak latency of mean waveform of a neuron. (a) Each dot corresponds to one unit from BF (green), OFC (red), V2 (blue) overlapped with putative excitatory (+) and inhibitory (o) markers. Right marginal distribution of burst index sorted by structures (BF green), (OFC red), (V2 blue). (b) examples of autocorrelogram and cross-correlogram of two putative excitatory cells and one inhibitory cell. First row from OFC (#73-87), second row from V2 (#2-#1) and third row from BF (#59-#54) with their waveforms. Scatter plot (c) green o- BF, (d) red o- OFC, (e) blue o- V2 superimposed with filled markers which correspond to the results of k-means clustering (six clusters) using the first (x-axis), and second (y-axis) W-PCA means second first and second PCA of second derivate waveform correspond to each of units (dots) with their TP latency (z-axis). (f) confusion matrix, x-axis corresponds to prediction results, y-axis shows the true class. (g) distribution of cells within k-means group with classified cholinergic cells marker (X), (h) mean of TC-latency grouped by k-means from BF, OFC and V2. (i) mean of firing rate grouped by k-means clustering. Each color represents the same cluster between (f), (g), (h), (i) subplots. Overall, the waveform of cholinergic cells falls into the wide-waveform excitatory group, and they are predictable based on their physiological features using the trained decision tree matching learning model. Thus, we can conclude that the [file 429_2021_2290_MOESM4_ESM.tif]
