# Supplementary material for: Contribution of the basal forebrain to corticocortical network interactions
Source: Brain Struct Funct. 2021 May 22;226(6):1803–21. doi: 10.1007/s00429-021-02290-z (PMC8203523; doi:10.1007/s00429-021-02290-z)
Supplement: Supplementary file 6 — Supplementary file6 (DOCX 23 kb) [file 429_2021_2290_MOESM6_ESM.docx]

| Behavioral epoch | Behavioral epoch | Mean Difference | 95% Conf. Interval | Sig. (p-values) |
| --- | --- | --- | --- | --- |
| cue presentation | doors opened | 0.00607 | (-0.11, 0.12) | *0.999* |
| cue presentation | decision making | -0.14539 | (-0.26, -0.02) | *0.011* |
| cue presentation | reward collection | -0.06383 | (-0.17, 0.04) | *0.538* |
| cue presentation | return | 0.010594 | (-0.10, 0.12) | *0.999* |
| cue presentation | optic stimulation | -0.10979 | (-0.22, 0.001) | *0.049* |
| doors opened | decision making | -0.15146 | (-0.28, -0.02) | *0.011* |
| doors opened | reward collection | -0.0699 | (-0.18, -0.04) | *0.498* |
| doors opened | return | 0.004525 | (-0.11, 0.12) | *0.999* |
| doors opened | optic stimulation | -0.11586 | (-0.23, 0.001) | *0.056* |
| decision making | reward collection | 0.081558 | (-0.04, 0.20) | *0.383* |
| decision making | return | 0.155984 | (0.02, 0.28) | *0.006* |
| decision making | optic stimulation | 0.035597 | (-0.08, 0.15) | *0.960* |
| reward collection | return | 0.074426 | (-0.03, 0.18) | *0.392* |
| reward collection | optic stimulation | -0.04596 | (-0.15, 0.06) | *0.826* |
| return | optic stimulation | -0.12039 | (-0.23, -0.006) | *0.032* |

**Table S2.a Multiple comparison using Tukey HSD test for BF-OFC relative coherence change at low gamma band**

| Behavioral epoch | Behavioral epoch | Mean Difference | 95% Conf. Interval | Sig. (p-values) |
| --- | --- | --- | --- | --- |
| cue presentation | doors opened | 0.00607 | (-0.11, 0.12) | *0.379* |
| cue presentation | decision making | -0.14539 | (-0.26, -0.02) | *0.041* |
| cue presentation | reward collection | -0.06383 | (-0.17, 0.04) | *0.962* |
| cue presentation | return | 0.010594 | (-0.10, 0.12) | *0.999* |
| cue presentation | optic stimulation | -0.10979 | (-0.22, 0.001) | *0.011* |
| doors opened | decision making | -0.15146 | (-0.28, -0.02) | *0.968* |
| doors opened | reward collection | -0.0699 | (-0.18, -0.04) | *0.808* |
| doors opened | return | 0.004525 | (-0.11, 0.12) | *0.457* |
| doors opened | optic stimulation | -0.11586 | (-0.23, 0.001) | *0.902* |
| decision making | reward collection | 0.081558 | (-0.04, 0.20) | *0.221* |
| decision making | return | 0.155984 | (0.02, 0.28) | *0.065* |
| decision making | optic stimulation | 0.035597 | (-0.08, 0.15) | *0.999* |
| reward collection | return | 0.074426 | (-0.03, 0.18) | *0.980* |
| reward collection | optic stimulation | -0.04596 | (-0.15, 0.06) | *0.089* |
| return | optic stimulation | -0.12039 | (-0.23, -0.006) | *0.020* |

**Table S2.b** Multiple comparison using Tukey HSD test for BF-OFC relative coherence change at high gamma band

| Behavioral epoch | Behavioral epoch | Mean Difference | 95% Conf. Interval | Sig. (p-values) |
| --- | --- | --- | --- | --- |
| cue presentation | doors opened | 0.0563 | (-0.055, 0.12) | *0.999* |
| cue presentation | decision making | 0.0159 | (-0.07, -0.12) | *0.978* |
| cue presentation | reward collection | -0.018 | (-0.17, 0.16) | *0.671* |
| cue presentation | return | -0.0818 | (-0.05, 0.14) | *0.895* |
| cue presentation | optic stimulation | -0.0361 | (-0.06, 0.09) | *0.998* |
| doors opened | decision making | -0.0404 | (-0.12, -0.09) | *0.999* |
| doors opened | reward collection | 0.0743 | (-0.08, -0.13) | *0.902* |
| doors opened | return | -0.0255 | (-0.07, 0.12) | *0.992* |
| doors opened | optic stimulation | 0.0924 | (-0.13, 0.07) | *0.043* |
| decision making | reward collection | -0.0339 | (-0.06, 0.12) | *0.943* |
| decision making | return | 0.0659 | (-0.08, 0.10) | *0.998* |
| decision making | optic stimulation | -0.052 | (-0.14, 0.05) | *0.854* |
| reward collection | return | 0.0998 | (-0.12, 0.08) | *0.997* |
| reward collection | optic stimulation | -0.0181 | (-0.18, 0.036) | *0.407* |
| return | optic stimulation | -0.1179 | (-0.16, 0.051) | *0.048* |

**Table S3.a** Multiple comparison using Tukey HSD test for BF-V2 relative coherence change at low gamma band.

| Behavioral epoch | Behavioral epoch | Mean Difference | 95% Conf. Interval | Sig. (p-values) |
| --- | --- | --- | --- | --- |
| cue presentation | doors opened | 0.094 | (-0.01, 0.20) | *0.136* |
| cue presentation | decision making | 0.039 | (-0.06, 014) | *0.878* |
| cue presentation | reward collection | 0.110 | (-0.01, 0.22) | *0.0464* |
| cue presentation | return | 0.029 | (-0.07, 0.13) | *0.9692* |
| cue presentation | optic stimulation | 0.011 | (-0.10, 0.12) | *0.999* |
| doors opened | decision making | -0.054 | (-0.14, 0.03) | *0.526* |
| doors opened | reward collection | 0.016 | (-0.08, 0.11) | *0.997* |
| doors opened | return | -0.064 | (-0.16, 0.03) | *0.411* |
| doors opened | optic stimulation | -0.082 | (-0.18, 0.02) | *0.208* |
| decision making | reward collection | 0.070 | (-0.02, 0.16) | *0.234* |
| decision making | return | 0.009 | (-0.09, 0.07) | *0.999* |
| decision making | optic stimulation | -0.027 | (-0.12, 0.06) | *0.962* |
| reward collection | return | -0.080 | (-0.17, 0.01) | *0.173* |
| reward collection | optic stimulation | -0.098 | (-0.20, 0.005) | *0.014* |
| return | optic stimulation | -0.018 | (-0.11, 0.08) | *0.996* |

**Table S3.b** Multiple comparison using Tukey HSD test for BF-V2 relative coherence change at low gamma band.
